# Use of genome-scale metabolic reconstructions of avian pathogenic Escherichia coli (APEC) phylogroups for the identification of lineage-specific metabolic pathways

**DOI:** 10.1099/mgen.0.001490

**Published:** 2025-09-17

**Authors:** Huijun Long, Jai W. Mehat, HuiHai Wu, Arnoud H. M. van Vliet, Roberto M. La Ragione

**Affiliations:** 1 Department of Comparative Biomedical Sciences, School of Veterinary Medicine, Faculty of Health and Medical Sciences, University of Surrey, Guildford, UK; 2 Discipline of Microbes, Infection and Immunity, School of Biosciences, Faculty of Health and Medical Sciences, University of Surrey, Guildford, UK; 3 Bioinfopipe Ltd, London, UK

**Keywords:** 3-hydroxyphenylacetate (3-HPAA), avian pathogenic *Escherichia coli* (APEC), *in silico* genome-scale metabolic model, pathotype

## Abstract

Avian pathogenic *Escherichia coli* (APEC) are a genetically diverse pathotype primarily associated with extra-intestinal infections in birds. APEC lineages are predicted to have unique metabolic capabilities contributing to virulence and survival in the host environment. Here, we present a genome-scale metabolic model for the APEC pathotype based on 114 APEC genome sequences and lineage-specific models for the phylogroups B2, C and G based on a representative isolate for each phylogroup. A total of 1,848 metabolic reactions were predicted in the 114 APEC isolates before gap filling and manual correction. Of these, 89% represented core reactions, whilst the 11% accessory reactions were mostly associated with carbon and nitrogen metabolism. Predictions of auxotrophy were confirmed by inactivation of the conditionally essential *lysA* and the non-essential *potE* genes. The APEC metabolic model outperformed the *E. coli* K-12 *i*JO1366 model in the Biolog Phenotypic Array platform. Sub-models specific to phylogroups B2, C and G predicted differences in the metabolism of 3-hydroxyphenylacetate (3-HPAA), a phenolic acid derived from the flavonoid quercetin, which is commonly added to poultry feed. Two 3-HPAA-associated reactions/genes distinguished APEC phylogroup C from APEC phylogroups B2 and G, and 3-HPAA supported the growth of APEC phylogroup C in minimal media, but not phylogroups B2 and G. In conclusion, we have constructed genome-scale metabolic models for the three major APEC phylogroups B2, C and G and have identified a metabolic pathway distinguishing phylogroup C APEC. This demonstrates the importance of lineage- and pathotype-specific metabolic models when investigating genetically diverse microbial pathogens.

## Data Summary

All genome sequencing data (sequencing reads and genome assemblies) are available at the Sequence Read Archive (SRA) and Genome repositories at the NCBI, in BioProject number PRJNA523197. The information on the individual APEC isolates has been included in Supplementary Material 2 (Table S1, available in the online Supplementary Material), whereas data on gene essentiality, Biolog measurements and model growth simulations have been included in Supplementary Material 2 (Tables S2–S4), respectively. The reactions included in the APEC GEM and phylogroup-specific sub-models, the media pfile versions for sub-models, carbon, sulphate, phosphate and the *lysA* and *potE* mutants have been made available via Figshare (https://doi.org/10.6084/m9.figshare.28067993), with the content of these files included as Supplementary Material 3–5 (Tables S5–S7), respectively.

**Impact Statement**

Avian pathogenic *Escherichia coli* (APEC) are the cause of colibacillosis in poultry, which results in a significant economic burden to the poultry industry and strongly affects the health and welfare of flocks. APEC isolates show a high level of genetic diversity, which complicates diagnostics, epidemiology and the design of prevention and treatment strategies. In this study, we have used genome sequences derived from 114 APEC isolates to investigate their metabolic capabilities and define the metabolic diversity of APEC within a generalized APEC metabolic model and lineage-specific metabolic models. These models have been interrogated to find unique pathways that can be targeted for the development of anti-APEC treatments, and one such metabolic pathway was identified as a proof of principle. This approach shows great promise for the design of future strategies to prevent and deal with APEC infections and can be adapted to other genetically diverse microbial pathogens.

## Introduction

Avian pathogenic *Escherichia coli* (APEC) belong to the extra-intestinal pathogenic group of *E. coli* [1,2]. APEC cause extra-intestinal infections in birds, commonly termed colibacillosis, a collective name describing a range of systemic and localized syndromes such as pericarditis, perihepatitis, peritonitis and airsacculitis. Colibacillosis causes significant economic losses and is a major welfare concern in the poultry industry [3]. The APEC pathotype is defined by the diseases caused in birds, with *E. coli* isolated from extra-intestinal locations in birds with colibacillosis-associated diseases being candidate APEC, pending further characterization. APEC are found in all major *E. coli* lineages and phylogroups and encompass a range of serogroups and multilocus sequence typing (MLST) sequence types (STs) [4,7], and it is this genetic variation and imprecise definition which complicates the development and efficacy of control strategies, such as antimicrobial administration and vaccine development [6]. Therefore, an improved understanding of the biology and variation of this pathogen will be beneficial to the development of novel and effective control strategies.

It has been proposed that different pathogenic *E. coli* lineages, in addition to commensal lineages, harbour variable metabolic profiles [8,10]. However, there remains a paucity of knowledge regarding the unique metabolic traits of APEC isolates, as most studies have focused on the identification of markers allowing distinction of APEC from avian faecal *E. coli* [7]. Genome-scale metabolic reconstructions (GEMs) are a powerful tool that has been applied in a number of fields [11]. Essentially, GEMs use genome sequences and database comparisons to predict metabolic functions and responses in a range of environmental conditions. To date, most *E. coli* GEMs have been constructed from a single commensal isolate, such as K-12 MG1655 [12,15], probiotic isolate [16,16] and *E. coli* W (ATCC 9637) [17]. However, as increasing numbers of *E. coli* genome sequences are now available, it has become apparent that GEMs of single commensal isolates only partially represent the metabolic capability of this species and its different pathotypes [9]. Similarly, it was reported that GEMs based on multiple isolates were able to differentiate the metabolic profile of pathogenic from commensal *E. coli* isolates [9]. Hence, it is clear that single laboratory strain-based models are not adequate to represent the metabolic capability and variation within *E. coli* pathotypes.

In this study, we have constructed a comprehensive metabolic genome-scale model based on a panel of 114 APEC isolates. This general APEC model was then used as a template to develop specific models that recapitulate the metabolism of the major phylogroups of the APEC pathotype. Models were queried for auxotrophy and gene essentiality and growth predictions in diverse media and used to identify a metabolic pathway distinguishing phylogroup C APEC from phylogroup B2 and G APEC, demonstrating the potential for such GEMs to improve our understanding of APEC biology and identification of potential targets for future prevention and intervention strategies.

## Methods

### Bacterial strains and growth conditions

One hundred and fourteen APEC isolates were obtained from the SAP culture collection at the School of Veterinary Medicine, University of Surrey, and used for APEC model construction (Supplementary Material 2, Table S1). These APEC isolates were obtained from commercial laying hens or broilers with either colibacillosis, perihepatitis, yolk sac infection, pericarditis or peritonitis in the UK and Germany. Isolates were collected at *post-mortem* examination per the protocols used at the diagnostic services involved and were submitted to the School of Veterinary Medicine of the University of Surrey between 2014 and 2017 for further diagnostic or research activity. The clinical information available is provided in Supplementary Material 2, Table S1. All isolates were stored in Brain Heart Infusion broth supplemented with 50% glycerol at −80 °C until required for analysis. Prior to analysis, the APEC isolates were streaked onto MacConkey agar 3 plates, nutrient agar or Luria–Bertani (LB) and incubated aerobically at 37 °C for 16–24 h, unless otherwise stated. M9 minimal medium (1×M9 salt, 2 mM MgSO_4_, 0.1 mM CaCl_2_ and 0.4% glucose if required) was used for specific experiments as indicated below. Chloramphenicol (Cm) and ampicillin (AMP) were used at final concentrations of 100 and 30 µg ml^−1^, respectively. All growth media and antibiotic supplements were purchased from Oxoid (Thermo Fisher Scientific) unless otherwise stated.

### Biolog Phenotypic Microarray and M9 minimal medium test supplemented with 3-hydroxyphenylacetate

Three APEC isolates were selected from the representative phylogroups B2, C and G (serogroups O2, O78 and O24, respectively) and used for Biolog analysis (*N*=3). Additional isolates from these phylogroups were selected for M9 minimal medium cultivation analysis (*N*=13). The isolates used for these analyses are listed in Supplementary Material 2, Table S1. Three biological and three technical replicates were performed for each isolate. The APEC isolates were pre-cultured on R2A agar plates (Biolog) for Biolog assays as per the manufacturer’s instructions [18] and subsequently tested using 190 carbon sources (PM1 and PM2 MicroPlate™), as well as 59 Phosphorus and 35 Sulphur Sources (PM4A MicroPlate™). The cell suspensions and plate inoculation were undertaken according to the instructions from the manufacturer. The Biolog PM1, PM2 and PM4A microplates were incubated and analysed in Biolog’s OmniLog® instrument at 37 °C for 24 h, aerobically. Readings were taken every 15 min. M9 minimal media was used to test the metabolic ability of isolates in a defined synthetic medium. Three concentrations (1, 0.75 and 0.5 mM) of 3-hydroxyphenylacetate (3-HPAA) were tested in this study. l-Lysine or putrescine (1 mM) was also tested in this study for the cultivation analysis of gene knockout mutants. The polystyrene 96-well plates (Thermo Fisher Scientific) were cultured and read in Spark® microplate reader (TECAN) at 37 °C for 24 h, aerobically. Readings were taken every 15 min.

### DNA extraction and next-generation sequencing

The high molecular weight genomic DNA of APEC isolates was extracted and purified using a DNA Wizard® genomic purification kit (Promega), according to the instructions from the manufacturer. The extracted DNA samples were sent to the Animal and Plant Health Agency (Weybridge, UK) for sequencing on the Illumina MiSeq platform, providing 150 nt paired-end reads. The obtained raw DNA sequences were *de novo* assembled in contigs using Shovill version 1.1.0 (https://github.com/tseemann/shovill) using version 3.14 of the Spades assembler [19] with minimum coverage of 2.00× and minimum length of 200 nt. Quality assessment of genome assemblies was taken by QUAST version 5.0 [20] and required the following minimum metrics to be satisfied: genome size between 4.5 and 6.0 Mbp, number of contigs <500 and N_50_ values of >50 kb, respectively (Supplementary Material 2, Table S1). Prokka version 1.14 [21] was used for initial annotation of genome sequences, which produced relevant output files compatible for downstream analyses. The phylogenetic grouping was analysed on the *in silico* based ClermonTyping program [22]. *In silico* serotyping was performed using Abricate version 1.0.1 (https://github.com/tseemann/abricate) with the EcOH [23] database. MLST sequence typing was performed using the mlst version 2.23 program (https://github.com/tseemann/mlst) with the Warwick scheme [24]. A phylogenetic tree based on core genome single nucleotide polymorphisms was generated using ParSNP version 1.2 [25], resulting in a phylogenetic tree highlighting genomic similarity and sharing of core genome of all isolates. All 114 APEC genomes were screened for 11 proposed virulence markers [26] using Abricate. All relevant information for the 114 genomes (assembly metrics, presence/absence of virulence genes, O:H serotype information and MLST STs) is included in Supplementary Material 2, Table S1.

### Construction of APEC metabolic models

The FASTA files of 114 APEC samples generated from Prokka were submitted to the RAST server (https://rast.nmpdr.org/) for genome annotation [27]. The RAST annotation was automatically submitted to ModelSEED (https://modelseed.org/genomes/) for further model draft construction [28]. Model gap filling is an essential step to investigate the missing reactions, which apply a mixed-integer linear programme to define the minimal number of reactions required for the customer-defined collection of reactions. Several algorithms have been reported and applied in model gap filling [29,30]. COBRA Toolbox version 3.0 [31] was used in this study, and the database was based on ModelSeed. Further manual curation was performed when the model was unable to be corrected by gap-filling steps, for instance: unnecessary reactions must be on for model running, and to avoid stoichiometrically balanced cycles which are a subset of contiguous reactions that forms a loop or internal network reactions that carry fluxes without any exchange reactions involved. The model was adjusted based on the literature (basic nutrient requirement for cell growth and common pathways) and the reference model *i*JO1366 [14]. The correction procedure included adding/deletion of reactions, adjusting of reversibility/directionality of reactions and change of biomass consumption reactions. Metabolic models have been included as Supplementary Material 3 (Table S5), Supplementary Material 4 (Table S6) and Supplementary Material 5 (Table S7).

### GEM analysis

The growth prediction analysis was performed on the SurreyFBA 2.0 beta with JyMet2 [32]. The model was imported as a SurreyFBA formatted file; meanwhile, a specific format (pfile) was also introduced, in which the media setting can be adjusted via changing the upper/lower bound (lb) of each exchange reaction. The computational prediction of growth rate was based on typical flux units [millimole per gram dry weight per hour (mmol gDW^−1^ h^−1^)]. The default nutrient sources were glucose (carbon, C), ammonia (NH_3_), phosphate (PO_4_^2-^) and sulphate (SO_4_^2-^). When examining the usage of a particular nutrient source, the exchange reaction of its corresponding default source was adjusted to zero (reaction shut down). In addition, growth simulation studies were performed using the *E. coli* K-12 model *i*JO1366 [14] for comparative analysis. Due to the differences in default nutrient requirements in modelling, two growth simulations (*i*JO1366a and *i*JO1366b) were performed within two different input nutrient settings. The input nutrients of *i*JO1366a were adjusted as in the APEC model used here, where the exchange reactions of selenate (SeO_4_^2-^), selenite (SeO_3_^2-^) and tungstate (WO_4_^2-^) were disabled as they are absent from the APEC model, whilst these three exchange reactions were enabled in *iJO1366*b to perform the optimal conditions for *i*JO1366. In both *i*JO1366a and *i*JO1366b, the exchange reactions of molybdate (MoO_4_^2-^) and nickel (Ni^2+^), which are also absent from the APEC model, were kept enabled, as they are essential for *i*JO1366. The phenotypic and computational measurements were compared using a Pearson correlation coefficient test to measure the strength and direction of these two variables.

### Inactivation of genes in *E. coli*

Genes were inactivated using the Red Lambda Recombination assay [33]. Two gene targets (*lysA* and *potE*) were selected based on gene essentially predicted by the model. The target genes were disrupted by deletion of ~450–550 bp sequence in the middle part of the gene sequence and replaced with a Cm resistance cassette as selective marker. These cloning fragments were constructed using gBlocks gene fragments in tubes service (gBlocks™, Integrated DNA Technologies). APEC isolates were selected for gene inactivation experiments from phylogroups B2, G and C, respectively, with 90% coverage and identity for the *lysA* and *potE* genes and no resistance to AMP and Cm in Mueller–Hinton broth, as these were used as selective markers for the inactivation procedure and associated plasmids. Electrocompetent cells were generated by three times washing with ice-cold 10% glycerol and electroporated with the assistant plasmid pSIJ8 [33,34], containing an AMP resistance gene. The transformed cells with pSIJ8 were selected on LB agar supplemented with AMP, followed by PCR using primers described in Supplementary Material 2, Table S8. The expression of the pSIJ8-encoded λ-Red recombination system was induced with 0.5M arabinose. The cloning fragment was then introduced through electroporation, with cells recovered at 30 °C (as plasmid pSIJ8 is temperature sensitive) on a shaking platform for 1–2 h. Mutants were selected on Cm-containing LB plates and verified by PCR. We also designed a reverse transcription quantitative-PCR to confirm the absence of *lysA* and *potE* mRNA transcripts in mutant candidates. The primers and probe of the real-time quantitative PCR were designed to amplify the sequences deleted from the WT isolate during the construction of the cloning fragment (Supplementary Material 2, Table S8).

## Results

### Genomic characterization of APEC isolates for metabolic model generation

**Fig. 1.**
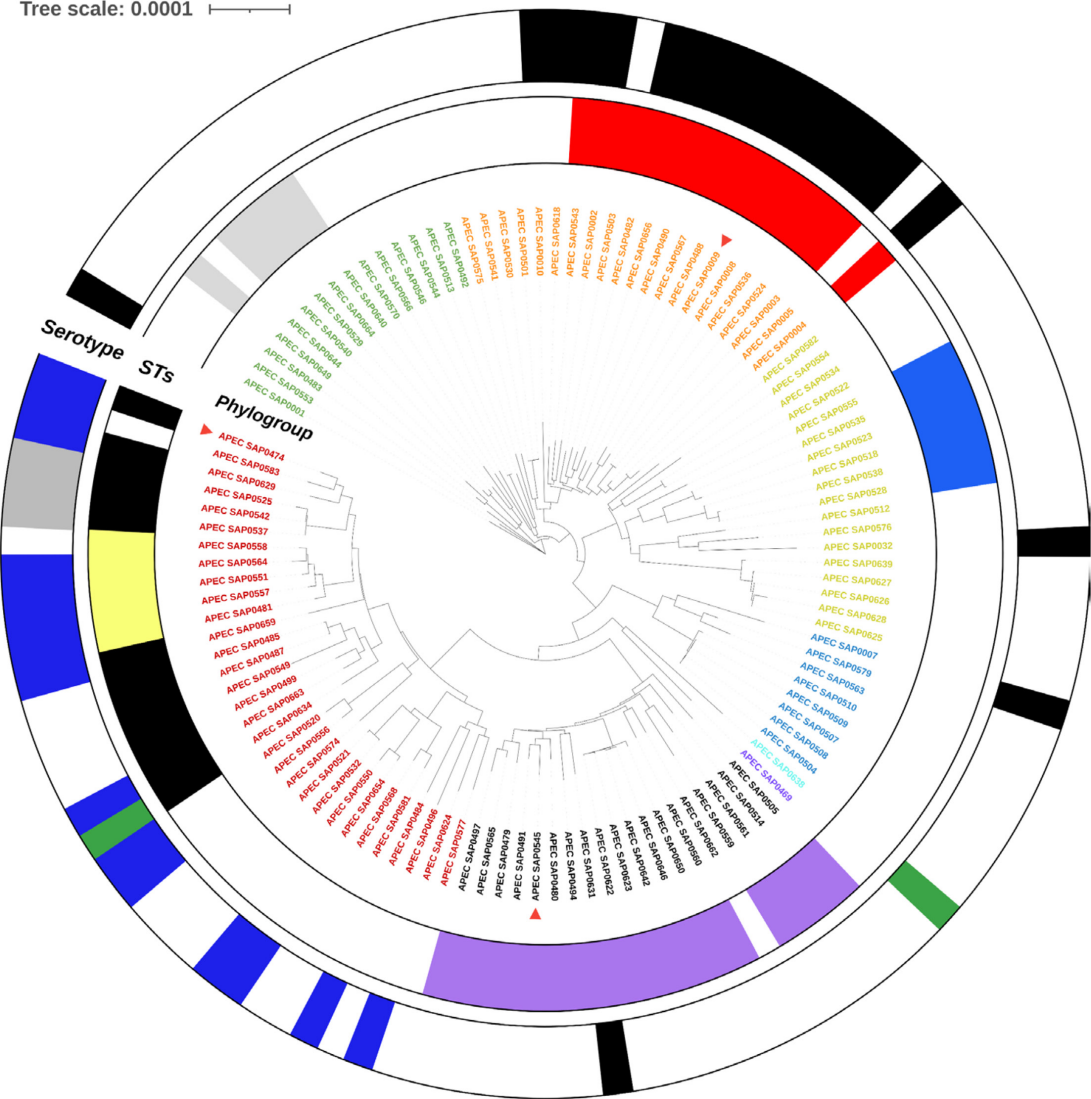
APEC population structure for the isolates selected for GEM construction in this study assigned with phylogroup (*n*=114). Colour code of phylogroups: A, yellow; B1, green; B2, red; C, orange; D, purple; E, blue; F, light blue; G, black. The distribution of dominant serogroups is shown in the outer ring. The colour code of serogroup: O78, black; O2, blue; O18ac, grey; O1, green. The distribution of dominant STs is shown in the middle ring. The colour code for STs: ST-117, purple; ST-23, red; ST-95, black; ST-93, blue; ST-101, grey; ST-140, yellow. The three representative APEC isolates from phylogroups B2, C and G used for Biolog phenotype assays are highlighted with a red triangle symbol.

A total of 114 *E. coli* isolates were included in this study, isolated from diseased birds from the UK and Germany. The pathologies observed are provided in Supplementary Material 1, Table S1, and ranged from colibacillosis, perihepatitis, peritonitis and pericarditis to yolk sac infection. To test whether these 114 APEC isolates were representative of the APEC pathotype, their genome sequences were determined using Illumina sequencing and subsequent genome assembly. The total size of the genome assemblies was between 4.72 and 5.56 Mbp (average 5.11 Mbp), with the number of contigs ranging from 104 to 327 (average 197) and the N_50_ ranging from 63 to 342 kbp (average 159 kbp). Subsequently, their phylogroups, dominant serogroups and STs were determined using the genome sequences (Fig. 1). The phylogenetic grouping of isolates based on core genome SNPs matched the distinctions in phylogroups and STs. *In silico* phylogroup typing showed that the majority of APEC isolates belonged to B2 (27.2%), followed by C (18.4%), G (16.7%), A (15.8%) and B1 (13.2%). The phylogroups D, E and F were underrepresented in this collection. The most prevalent serogroups were O78 (17%) and O2 (13%), and the most common MLST STs were ST-117 (15.8%), ST-23 (12.3%) and ST-95 (10.5%). Screening of the genome sequences for the presence of 11 virulence markers showed that 83/114 (73 %) of the included APEC isolates had 5 or more of these virulence markers. All these characteristics broadly matched earlier studies [4,7,35], and hence, we consider this collection of isolates broadly representative for the APEC pathotype.

### Generation of the metabolic profile of the APEC pathotype

**Fig. 2.**
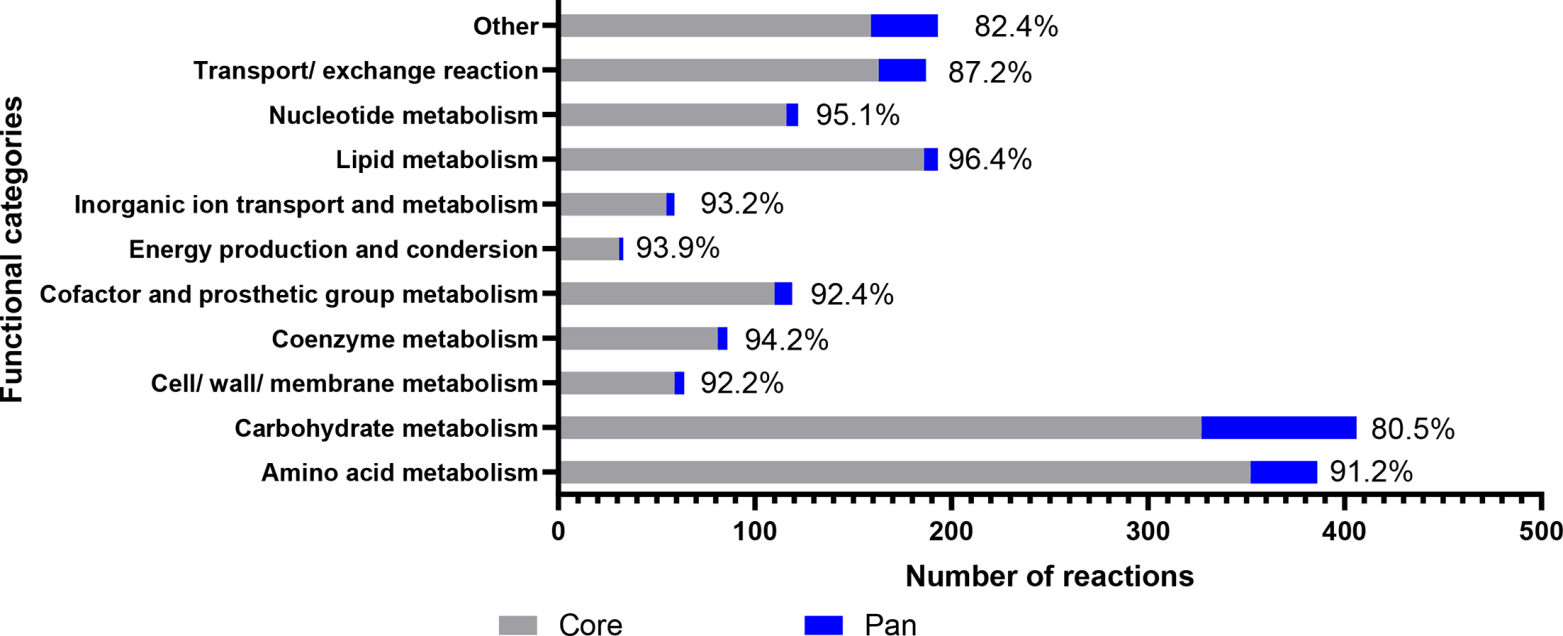
The distribution of annotated metabolic reactions screened by ModelSEED in 114 APEC isolates. This figure illustrates the core (grey) and pan (blue) metabolic content of the 114 APEC isolates used in this study. The core and pan content were defined by the extent of content shared among all isolates. The percentage shown represents the core reactions of the total.

**Fig. 3.**
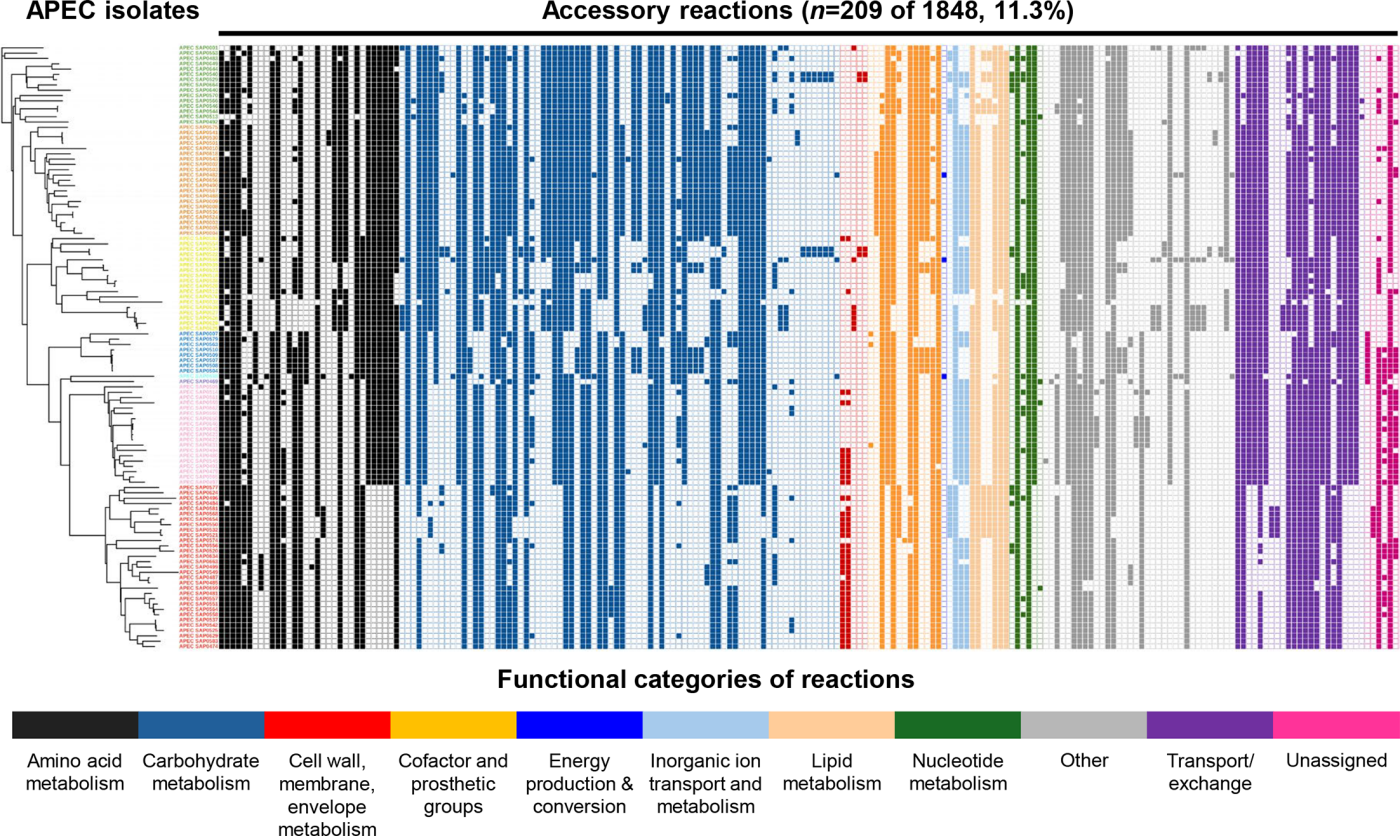
Distribution of accessory metabolic reactions in APEC (*n*=114) among different phylogenetic groups, clustered using a tree based on core genome single nucleotide polymorphisms. Colour code of phylogroups (from top to bottom): yellow, A; green, B1; orange, C; dark blue, E; purple, D; light blue, F; red, B2; pink, G. Presence of metabolic reactions is represented using a filled square and absence of the reaction by an open square. Only the 209 accessory reactions of the total of 1,848 reactions (pan) are shown here; a full overview of reactions and distribution in the 114 APEC is provided in Supplementary Material 2, Table S9. The functional categories of accessory reactions are also shown here. Colour code of functional categories of reactions (from left to right): black, amino acid metabolism; blue, carbohydrate metabolism; red, cell wall/ membrane/envelope metabolism; orange, cofactor and prosthetic group metabolism; dark blue, energy production and conversion; light blue, inorganic ion transport and metabolism; beige, lipid metabolism; green, nucleotide metabolism; grey, other; purple, transport/exchange reaction; dark pink, unassigned (*Unassigned reactions were not involved in reconstruction of APEC GEM or phylogroup-specific Sub-GEMs, as determined as dead-end reactions by gap filling).

The metabolic profile of the APEC isolates was predicted using ModelSEED. A total of 1,848 different metabolic reactions were identified and annotated in the 114 APEC isolates included and were defined as pan reactions. A total of 1,639 reactions (88.7%) were shared among all isolates, defined as core reactions, whereas 209 reactions (11.3%) were defined as accessory reactions. The reactions were categorized according to their predicted metabolic functions using ModelSEED, as shown in Fig. 2. The most highly conserved metabolic reactions were responsible for lipid metabolism (96.4%), nt metabolism (95.1%), coenzyme metabolism (94.2%) and energy production (93.9%). Reactions for alternative carbohydrate utilization and aa metabolism made up 42.9% (792/1,848) of pan reactions and 52.6% (110/209) of accessory reactions. The metabolic reactions were subsequently clustered with the phylogroups identified (Fig. 3, Supplementary Material 2, Table S9). In the accessory reactions, there was clustering of metabolic profiles of APEC isolates from the same phylogroups, especially in the metabolism of alternative carbohydrates and aa including their transport/exchange capabilities. This suggests that certain lineages are likely adapted to niches or environments, where they may harbour an advantage in the utilization of available nutrient. Notably, phylogroup B2 APEC isolates displayed distinct metabolic capabilities compared with the other phylogroups. Similar observations were also reported previously [36,37]. Metabolic capabilities absent in phylogroup B2 were mainly associated with aa and carbohydrates and related transport/exchange of metabolites. In contrast, metabolic reactions involved in membrane biogenesis, such as *N*-acetylneuraminic acid (Neu5Ac) metabolism, were predominantly clustered in phylogroups B2 and G. These reactions have been described to contribute to the pathogenicity of Gram-negative bacteria by protecting against the host immune system [38].

### Characterization of APEC GEM and phylogroup-specific sub-models

**Fig. 4.**
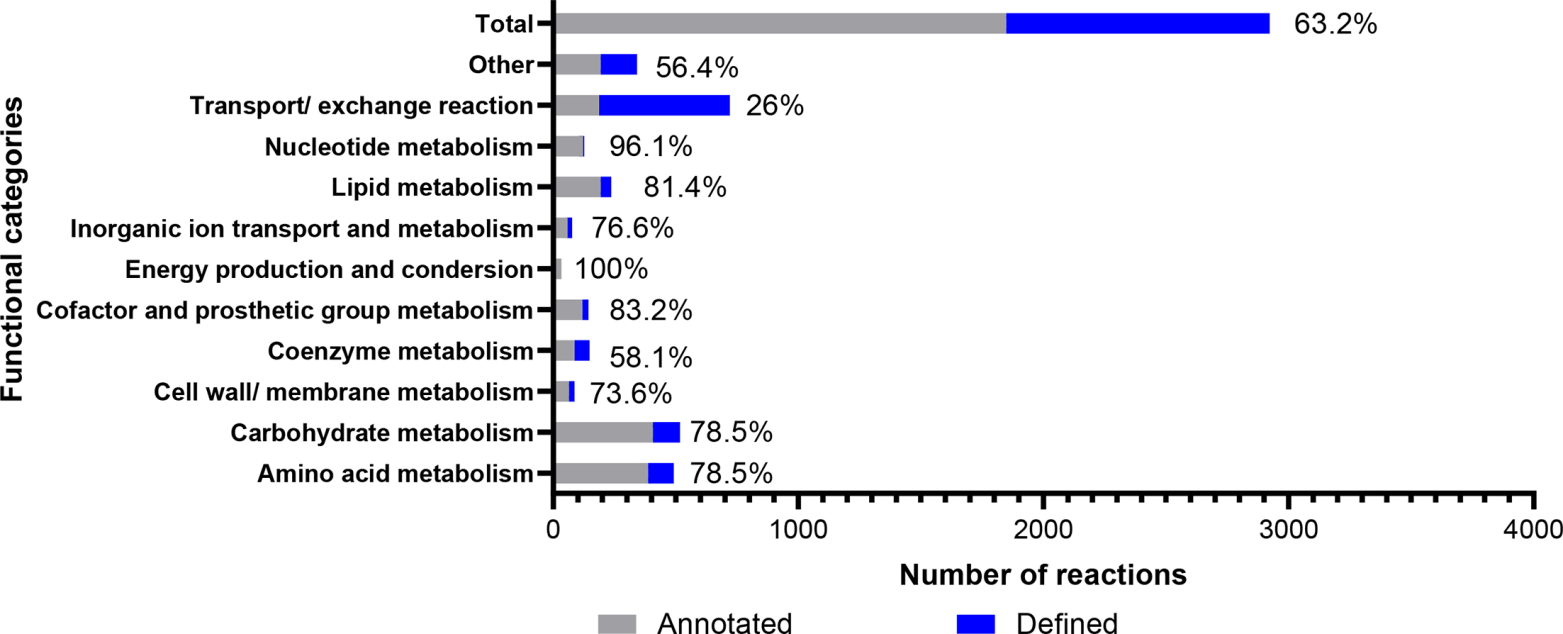
The distribution of the final defined reactions (grey) in the APEC model after gap filling and manual curation compared with the annotated metabolic reactions (blue) screened by ModelSEED in 114 APEC isolates. The percentage shown represents the fraction of reactions retained in the model.

The 1,848 metabolic reactions identified in the APEC isolates were used to generate a draft genome-scale metabolic model using ModelSEED. Sixteen reactions were excluded as dead-end reactions. Eventually, the pan-genome metabolic model constructed in this study, referred to as APEC GEM, was composed of 2,923 reactions (inclusive of gap-filling reactions and a total biomass reaction), 2,527 metabolites and 2,242 genes (Fig. 4). Notably, there were many reactions added during gap-filling algorithms and manual curation, such as spontaneous reaction for extracellular metabolite exchange, isomerization and putative reactions, which significantly change the distribution of functional categories compared with Fig. 2. Based on KEGG annotations and the *E. coli* K-12 model [14], a large number of exchange reactions of extracellular metabolites were accumulated in the transport/exchange functional category (Fig. 4). This contrasted with the central metabolism reactions, such as those involved in energy production and nt metabolism, which are generally conserved across *E. coli* lineages.

The APEC GEM was built as a pan-genome metabolic model, to represent the full metabolic capabilities in the complete APEC collection used in this study. Considering the distinct metabolic variances between different APEC lineages (Fig. 3, Supplementary Material 2, Table S9), phylogroup B2, C and G-specific models were generated from the APEC GEM to represent the metabolic capability and deficiency of each phylogroup. A brief summary of the reactions associated with different metabolic categories removed for sub-model construction for each phylogroup is shown in Supplementary Material 1, Fig. S1. In total, 133 reactions were deleted during the construction of sub-models. Notably, the same set of metabolic pathways was removed from more than one phylogroup. Most of the deleted reactions were associated with carbohydrate metabolism, accounting for 42%, followed by aa metabolism (14.3%) and transport/exchange reactions (9%). A relatively high ratio of carbohydrate (13/19) and aa (45/57) metabolism reactions and transport/exchange reactions (9/12) was removed in phylogroup B2 in this study, consistent with prior observations. Besides, 39/57 of carbohydrate metabolism reactions were removed for phylogroup G.

### Gene essentiality analysis of APEC GEM

Gene essentiality analysis is often adopted to evaluate the validity of GEM by comparing with experimentally obtained essential gene datasets [9,16]. The APEC GEM was used to predict growth on glucose and glycerol as carbon sources. For growth on glucose or glycerol, the lower boundaries of the glucose and glycerol exchange reactions were set to 1,000, respectively. All 2,242 genes in APEC GEM were inactivated *in silico* individually as time and growth were simulated by flux balance analysis (FBA) (Supplementary Material 2, Table S2). When inactivation of a gene resulted in a growth rate above zero, it was considered as a non-essential gene. The gene essentiality predictions on glucose and glycerol in minimal media showed a consistent outcome using the APEC model in this study (Supplementary Material 2, Table S2). In general, *in silico* inactivation of 208 out of 2,242 genes (9.3%) led to an absence of flux through the network and as such would lead to growth inhibition and thus were determined to be essential genes in the APEC model. The distribution of these essential genes is shown in Supplementary Material 1, Fig. S2. Of these essential genes, 57 (27%) were associated with more than 1 reaction in this model. Thirteen per cent were responsible for aa metabolism and 3% for carbohydrate metabolism. To validate these predictions, we decided against the use of the Keio collection grown on glucose/glycerol, as there are significant differences between *E. coli* K-12 and APEC. Hence, two single-gene knockout mutants were constructed in APEC for this purpose, in the essential *lysA* gene and non-essential *potE* gene. Both genes were present in 100% of the 114 APEC isolates analysed in this study.

The *lysA* gene is predicted to encode a diaminopimelate decarboxylase that is responsible for the biosynthesis of lysine. In the model, the *lysA* gene (model ID: peg18487) was determined to be an essential gene and encoded for a chemical equation (model ID: R_rxn00313) as below ([c] represents the metabolites in the cytoplasm):

Rxn00313: (1) H^+^ [c]+(1) meso-2,6-diaminopimelate [c] => (1) CO_2_ [c]+(1) L-Lysine [c]

The *potE* gene encodes a membrane protein mediating both the uptake (via proton symport mechanism) and excretion of putrescine (as a putrescine and ornithine antiporter). There is redundancy in putrescine transport with the PotABCD, PotFGHI, PlaP/YeeF, PuuP and SapBCDF systems [39,42]. In the model, the target gene *potE* (model ID: peg6625) was determined as non-essential and responsible for two putrescine transport reactions (model ID: R_rxn05867 and R_rxn10182). For the modelling purpose, the chemical equations of these two reactions are represented as below ([e] represents the extracellular metabolites; [c] represents the metabolites in the cytoplasm):

Rxn05687: (1) H^+^[e] + (1) putrescine[e] => (1) H^+^[c] + (1) putrescine[c]

Rxn10182: (1) ornithine[c] + (1) putrescine[e] <=> (1) ornithine[e] + (1) putrescine[c]

**Fig. 5.**
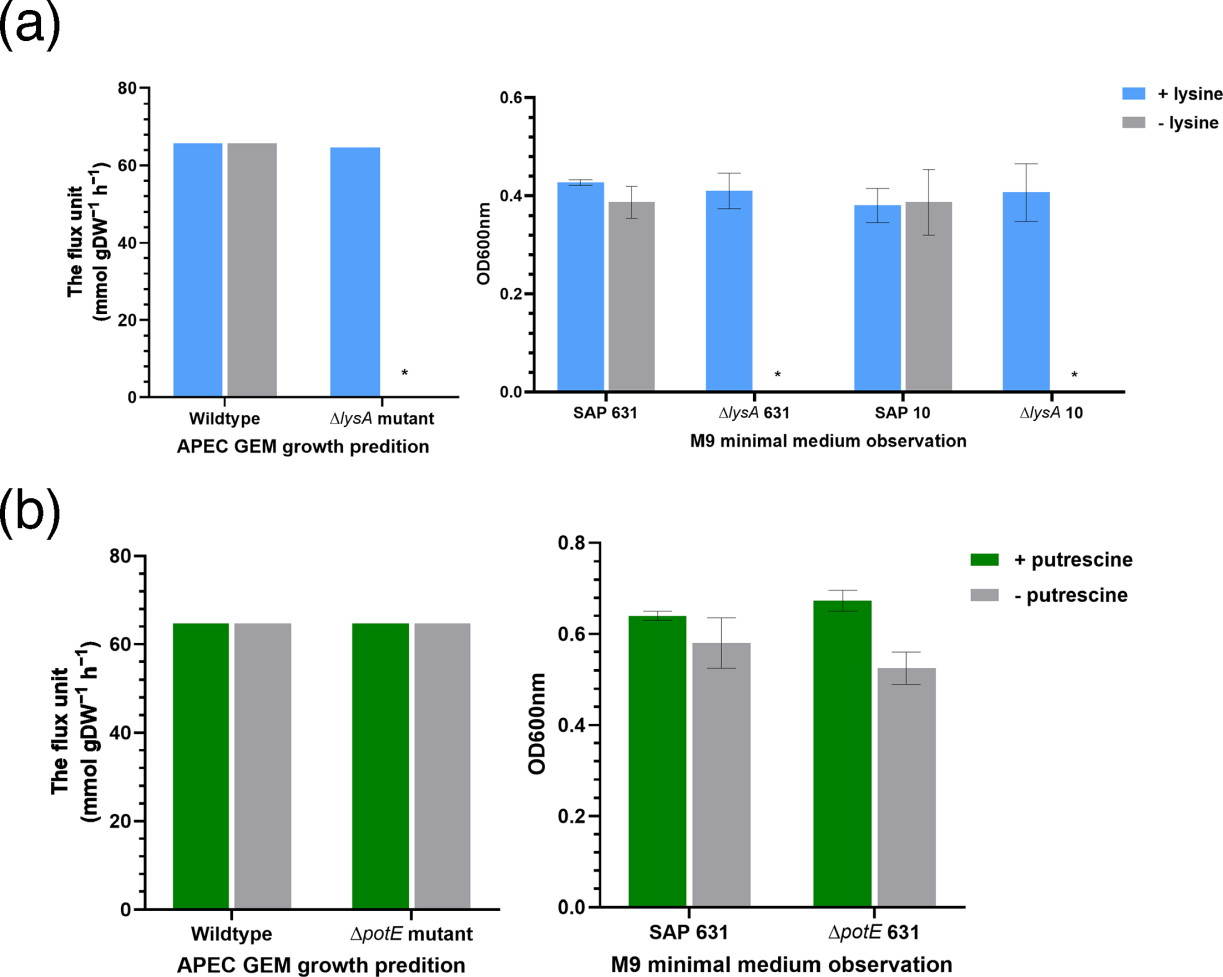
The computational growth prediction (left) and phenotypic observation (right) of WT and single-gene knockout isolates under constrained nutrient input. (a) shows the effect of inactivation of *lysA* with/without medium supplementation with lysine; an asterisk indicates lack of growth, demonstrating the essential role of the *lysA* gene. (b) shows the effect of inactivation of *potE* with/without medium supplementation with putrescine, which had no effect as the gene is not essential. The computational prediction is generated by APEC GEM, and data are measured by flux unit (mmol gDW^−1^ h^−1^). The phenotypic observation is measured using M9 minimal medium, and the OD is recorded after 24-h incubation (37 °C) at a wavelength of 600 nm in a 96-well plate. The error bar shows sd of the mean of three biological replicates. Asterisks indicate an absence of growth.

The *lysA* mutants generated in isolates SAP0010 and SAP0631 and *potE* mutant generated in isolates SAP0631 were analysed using glucoside as the default carbon source. Other default nutrient sources were adjusted by the general standard principle described in the next section. The *in silico* growth prediction of knockouts grown with and without supplementation of l-lysine or putrescine was compared with the phenotypic screening using M9 minimal medium as illustrated in Fig. 5. The ∆*lysA* mutants (∆*lysA* 10-9 and ∆*lysA* 631-11) were observed to be auxotrophic for l-lysine (Fig. 5), which indicates the fact that the *lysA* gene encodes the enzyme in the final and only lysine biosynthesis pathway. This was also revealed in APEC GEM knockout analysis that no biomass was produced when the rxc00313 was switched off. On the contrary, the supplementation of putrescine in M9 medium did not lead to any significant change in growth between the ∆*potE* 631–12 mutant and its parental strain (Fig. 5). The APEC GEMs correctly predicted the growth status of these knockout mutants, as well as their WT, in minimal media with and without supplementation.

### Comparison of the APEC GEM with the *E. coli* K-12 *i*JO1366 GEM

**Fig. 6.**
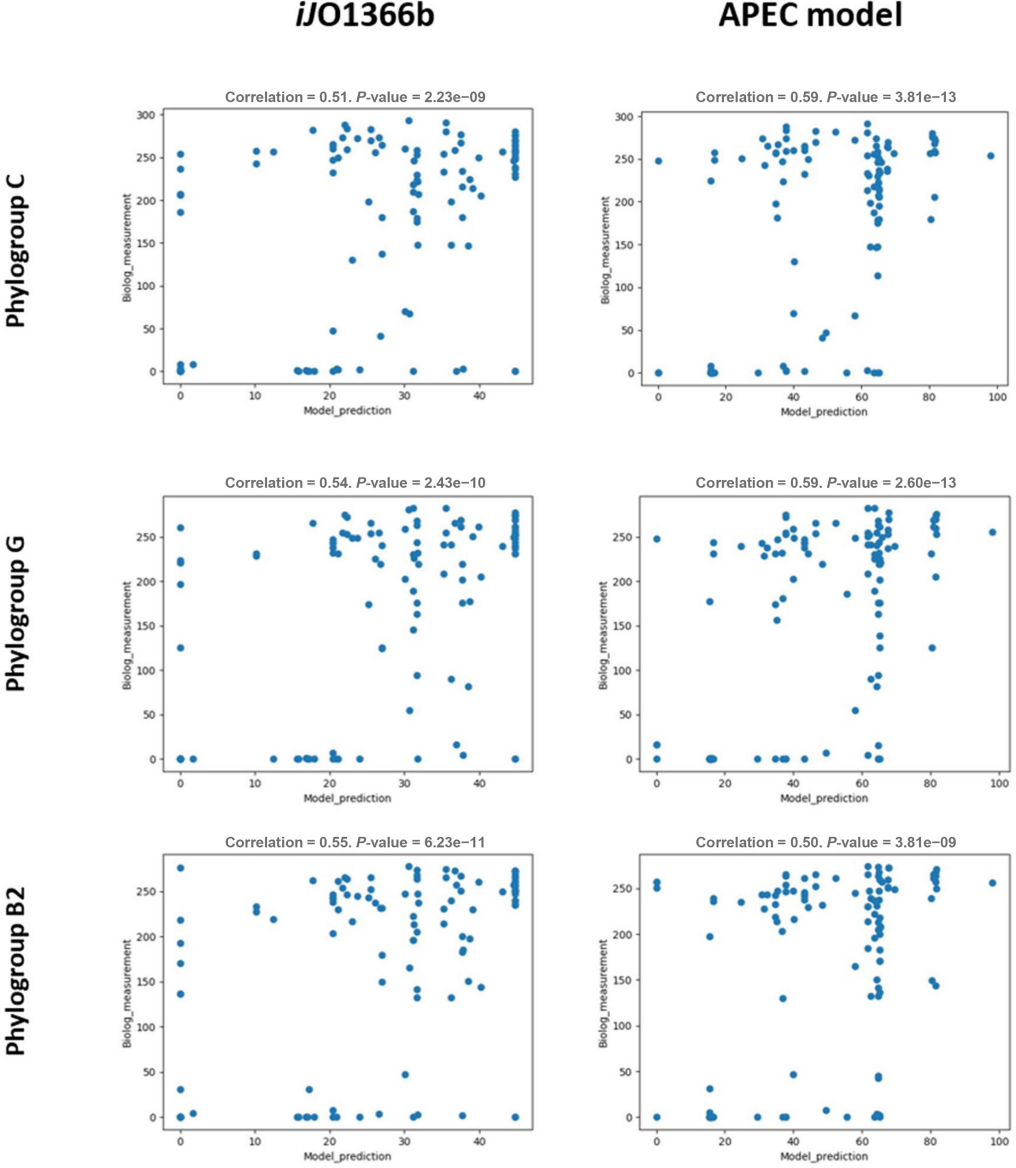
Scatter plots comparing computational predictions from the *E. coli* K-12 *i*JO1366 model and the APEC model for three APEC isolates representing phylogroups B2, C and G, with Biolog phenotypic observations with single nutrient source utilization. The APEC isolates used are from phylogroups C (SAP0009), G (SAP0545) and B2 (SAP0474). The scatter plots show the model prediction (x-axis) using *i*JO1366b (left) and APEC model (right) and phenotypic observation using Biolog measurement (y-axis). The correlation value shown above graphs is the Pearson correlation coefficient *r*^2^ value.

To validate *in silico* model prediction with phenotypic observations, the growth (respiration) phenotype of three representative APEC isolates, chosen from phylogroups B2, C and G, was tested using the Biolog Phenotypic Microarray analysis (PM1, PM2A: carbon source plates and PM4A: sulphate and phosphate plate). The respiration measurements are available in Supplementary Material 2, Table S3. In total, 125 nutrient substrates were analysed in the APEC model, and 119 substrates were analysed in *i*jO1366 (Supplementary Material 2, Table S4). The Pearson correlation coefficient of all three comparison experiments showed mostly positive correlations; the *i*JO1366a model showed a weak positive correlation (*r*^2^ values between 0.07 and 0.17, Supplementary Material 1, Fig. S3), while the *i*JO1366b and APEC models showed moderate positive correlations (*r*^2^ values between 0.50 and 0.59, Fig. 6). Under the optimized media settings, the APEC model showed an improved performance with the representative phylogroups C (*r*^2^=0.59, *P*<0.005) and G (*r*^2^=0.59, *P*<0.005), while it showed decreased performance with phylogroup B2 (*r*^2^=0.50, *P*<0.005), compared with model *i*JO1366 (Fig. 6).

### Comparison of the APEC GEM with phylogroup-specific sub-models

**Fig. 7.**
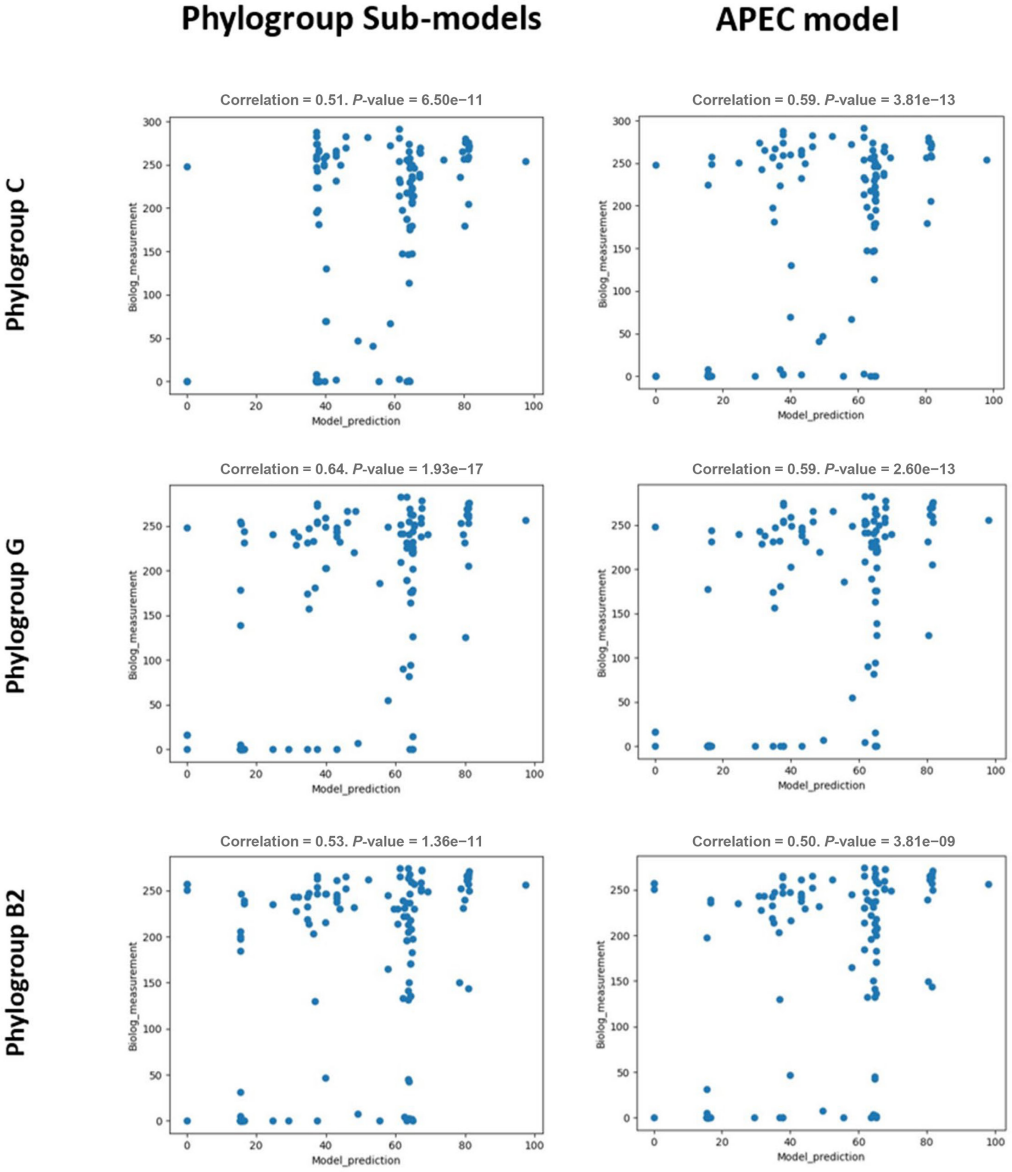
Scatter plots comparing computational predictions from the APEC phylogroup-specific sub-models for phylogroups B2, C and G with the general APEC model for three APEC isolates representing phylogroups B2, C and G, with Biolog phenotypic observations with single nutrient source utilization. The APEC isolates used are from phylogroups C (SAP0009), G (SAP0545) and B2 (SAP0474). The scatter plots show the model prediction (x-axis) using *i*JO1366b (left) and APEC model (right) and phenotypic observation using Biolog measurement (y-axis). The correlation value shown above graphs is the Pearson correlation coefficient *r*^2^ value.

We also explored the performance of phylogroup-specific APEC model-based sub-models. The default media setting of phylogroup-based sub-models was modified from the APEC GEM, with the import of Fe(II)-nicotianamine [Fe(II)-NA or Fe^2+^-NA] added as it was required for model performance of sub-models for phylogroups C and G. In total, *in silico* FBA of 142 nutrient substrates was performed with phylogroup-specific sub-models and then compared with growth simulation of APEC GEM according to Biolog data (Supplementary Material 2, Table S4). In Fig. 7, the relationship between the computational prediction from the sub-models and their Biolog measurements showed moderate positive correlation (*r*^2^ values of 0.51, 0.64 and 0.3 for phylogroups C, G and B2, respectively). Compared with the computational prediction by the pan model APEC GEM, the sub-models of phylogroup C performed less well under similar media input, while the phylogroup G and B2 sub-models showed improved performance compared with APEC model.

### Identification of 3-HPAA as a phylogroup C APEC-specific nutrient

During querying of the phylogroup-specific APEC GEMs, one of the reactions predicted to be specific for phylogroup C APEC isolates was associated with the metabolism of 3- and 4-hydroxyphenylacetic acid:

Rxn02363: NADH+O_2_+H^+^+3-HPAA => H_2_O+NAD^+^+homoprotocatechuate

This reaction is catalysed by 4-hydroxyphenylacetate 3-monooxygenase oxygenase, encoded by gene *hpaB* (4-hydroxyphenylacetate 3-monooxygenase, EC 1.14.13.3). The *hpaB* gene was present in all phylogroup C (*n*=21/21) APEC, while absent in phylogroup B2 (*n*=0/31) and G (*n*=0/19) APEC. The *hpaB* gene was detected in phylogroups A and B1 as well, but these constitute minor phylogroups for APEC. The metabolic variation on 3-HPAA metabolism was measured using M9 minimal medium. As is shown in Supplementary Material 1, Fig. S4, five phylogroup C isolates were all able to grow with the three different concentrations of 3-HPAA tested, whereas six phylogroup B2 and two phylogroup G isolates were unable to grow with 3-HPAA as the single carbon source (Supplementary Material 1, Fig. S5), but all grew in LB medium.

The metabolism of 3-HPAA in *E. coli* has been described previously [43,44]. However, the intracellular/extracellular reaction of 3-HPAA in *E. coli* has not yet been documented in the ModelSEED database. Therefore, we manually added the exchange reaction of 3-HPAA into the sub-models, in order to perform the growth simulation with/without supplementary of 3-HPAA. The additional reactions are presented as below (cpd03320 is the compound ID of 3-HPAA in APEC GEM and relative sub-models, *e* represents extracellular; *b* represents periplasm; *c* represents cytoplasm):

R_e2c_cpd03320: M_cpd03320_e = M_cpd03320_c

R_EX_cpd03320: M_cpd03320_e = M_cpd03320_b

**Fig. 8.**
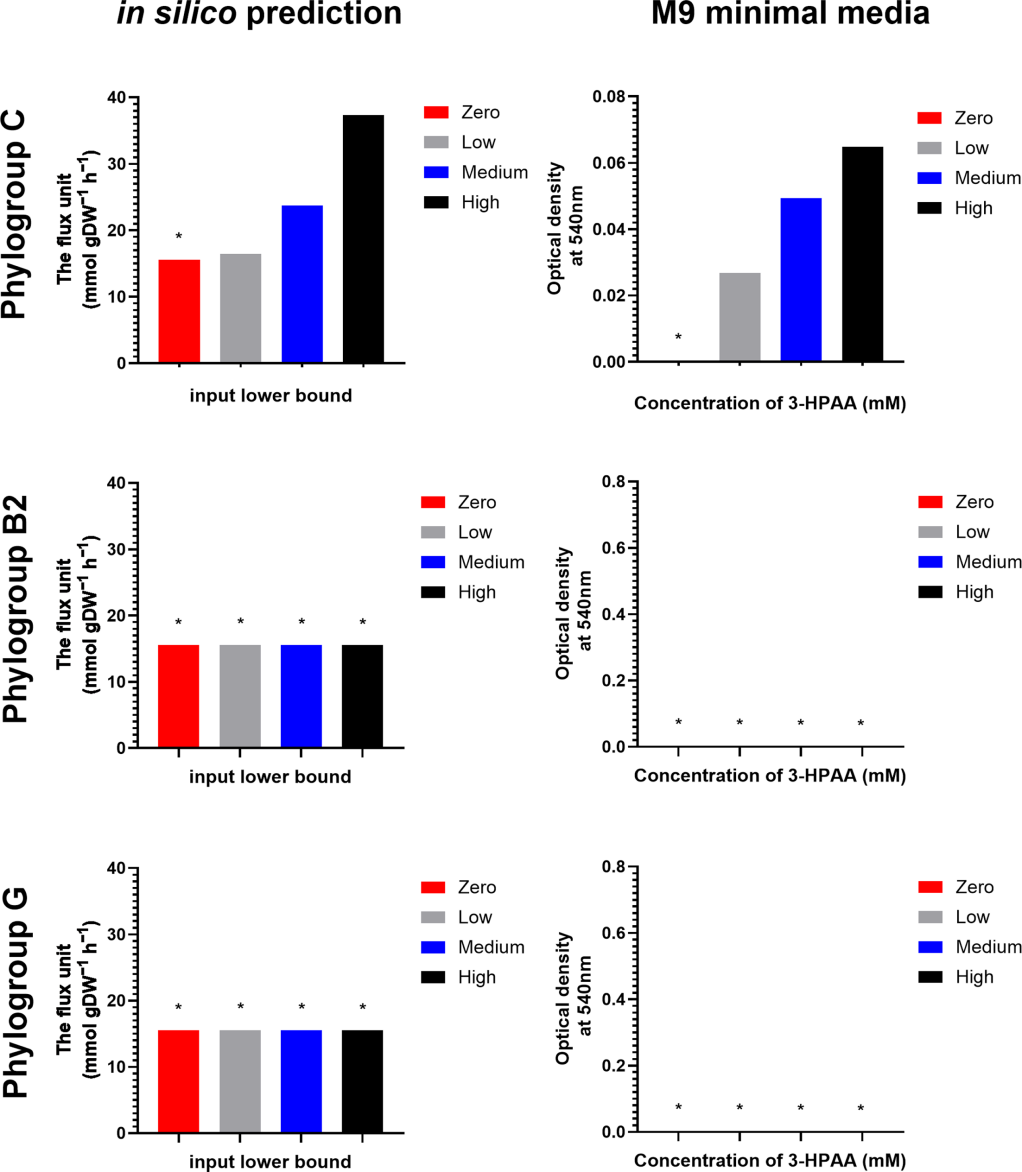
The comparison between *in silico* prediction and M9 minimal medium measurements of different phylogroup APEC isolates on the utilization of different concentrations of 3-HPAA. The left figures show the *in silico* prediction using phylogroup-based sub-model (C, G and B2) under the constraint of 3-HPAA exchange reaction lb adjusted to 0, –10 (low), −100 (medium) and −1,000 (high), respectively (−1,000 refers to unlimited uptake of inputs). The right figures show the phenotypic measurements in M9 minimal media with supplement of 0 mM (zero), 0.5 mM (low), 0.75 mM (medium) and 1 mM (high) 3-HPAA as the sole carbon source. The measurements indicate the mean of three biological endpoint (*t*=18 h) readings. More detailed growth curves are provided in Supplementary Material 1, Figs S4 and S5. Asterisks indicate an absence of growth.

The lb of the 3-HPAA exchange reaction was adjusted to 0, –10, −100 and −1,000 in the APEC GEM sub-models B2, C and G, to simulate APEC growth in response to four different concentrations of 3-HPAA. The growth prediction of different sub-groups and M9 experimental observation is summarized in Fig. 8. In sub-models B2 and G, the relatively minimal biomass (~15) was produced with the lb of 3-HPAA exchange reaction adjusted to 0, –10, −100 and −1,000 (increasing uptake), which was assumed as no growth for the modelling purpose. This agreed with the M9 minimal media experimental data, where isolates from phylogroup B2 and G were unable to use 3-HPAA as the sole carbon source. However, growth was predicted in sub-model C, matching the setting of increased uptake of 3-HPAA (Fig. 8).

## Discussion

In this study, we have investigated the metabolic capabilities of a representative set of 114 APEC isolates representing different phylogroups. A genome-scale metabolic model was constructed based on the genome sequences of these APEC isolates and used to (1) perform growth simulation experiments which were validated using phenotypic assays, (2) perform gene essentiality analysis, and (3) investigate functional difference between phylogroups and develop phylogroup-based sub-models.

The comparative genomic analysis performed in this study confirmed that the APEC pathotype is genetically variable, with phylogroups B2, C and G being the dominant lineages [4,5, 45]. The resulting genome-scale metabolic profile of the 114 APEC isolates (Fig. 3, Supplementary Material 2, Table S9) showed strong conservation in central metabolism involved in energy production, with the ratio of core metabolism versus accessory metabolism higher than in previous studies, where multiple *E. coli* pathotypes and commensal isolates were involved [9,46]. The major metabolic variances between isolates are associated with the metabolism of carbohydrates and aa, as well as exchange/transport reactions. This was confirmed using Biolog metabolic analysis performed in this study. As shown in Fig. 3 and Supplementary Material 2, Table S9, the phylogroup B2 isolates showed distinct metabolic features (mostly missing metabolic reactions) compared with others, which was previously also observed [36,47, 48]. This is consistent with the view that loss and acquisition of genes during evolution differentiates isolates into different lineages with unique metabolic profiles and adaptation to specific niches.

When comparing the predictions of the APEC model with the Biolog phenotypic measurements, the association between APEC GEM prediction and phenotypic observations was moderately positive (*r*^2^ values between 0.5 and 0.6). The vast numbers of reactions and genes encompassed within metabolic models potentially lead to a high risk of gaps or errors within the metabolic network. Furthermore, the reconstruction of GEMs is severely limited by the paucity of verified metabolic pathways that are specific to APEC and current knowledge in our database, including errors introduced by automatic annotation of genome sequences in the respective databases. From the perspective of APEC diversity, the pan-genome-scale metabolic model is too extensive to represent the differences and specifics of certain APEC lineages. Notably, in this study, we used the Biolog platform as the indicator of biomass formation of APEC isolates in response to different nutrient sources, while this assay is based on the measurement of microbial respiration and reflected by dye changes. These two different measurements may lead to false-positive or -negative results during the coefficient relationship analysis. This was addressed by changing the function from biomass production to indicator dye reduction used in the Biolog plates, which leads to a significant decrease in false-negative results [8]. Alternatively, a comprehensive screening of isolates using different nutrient sources by M9 minimal medium may provide more direct observation for model validation. Typically, the phenotype of the Keio collection was used in model validation of *i*JO1366 [14]. However, this was not appropriate for our study due to the significant differences between the laboratory strain K-12 and the APEC pathotype. Moreover, the APEC pathotype has a diverse metabolic profile and construction of a single-gene knockout library representing that the APEC pathotype is not possible, which made it difficult to apply the APEC metabolic model for the pathotype. However, the APEC metabolic model provides a comprehensive database of metabolic capabilities for a diverse panel of APEC and as such can be used for initial predictions and can be used as the basis for the construction of lineage-based strain sub-models, as performed here for the dominant phylogroups B2, C and G.

Although no significant improvement in the association strength between computational prediction of sub-models and the phenotypic measurement of the corresponding APEC isolates was observed, the APEC model showed its potential and plasticity as a pan model used for the construction of phylogroup B2, C and G sub-models. The phenotypic difference in catabolism of 3-HPAA in phylogroups B2, C and G was successfully predicted by those phylogroup-based sub-models as a proof of principle. Interestingly, 3-HPAA and 4-hydroxyphenylacetate are the main phenolic acids derived from flavonoid quercetin (which is commonly added to poultry feed) in the colorectum [49,50]. Therefore, these compounds are likely to accumulate in the avian intestine, and their utilization could be a hypothetical nutrient advantage in the intestinal microbial community. The phylogroup C sub-models not only predicted the growth of phylogroup C APEC isolates using 3-HPAA as the sole carbon source but also reflected the biomass increases alongside enhancing lb setting of 3-HPAA (Fig. 8, Supplementary Material 1, Fig. S4).

The models generated in this study can be further exploited for the identification of additional pathways that are unique to APEC lineages. Although APEC are extra-intestinal pathogens, potential APEC isolates are present in the intestinal *E. coli* population [7] and may form a reservoir for future infections within flocks. These models may inform the targeted suppression of such APEC populations, thereby benefitting avian health and welfare in the long term. The approach taken may also be applicable to other genetically diverse intestinal or extra-intestinal pathogens dispersed over multiple lineages. Furthermore, in the future, the rapid progress in software development (e.g. Bactabolize [51]), availability of APEC genome sequences and associated repositories may expedite the generation of genome-scale metabolic models to revisit or improve existing metabolic models for APEC and other *E. coli* pathotypes.

## Supplementary material

10.1099/mgen.0.001490 Uncited Supplementary Material 1.

10.1099/mgen.0.001490 Uncited Supplementary Material 2.

10.1099/mgen.0.001490 Uncited Supplementary Material 3.

10.1099/mgen.0.001490 Uncited Supplementary Material 4.

10.1099/mgen.0.001490 Uncited Supplementary Material 5.

## Abbreviations

AMP: ampicillin
APEC: avian pathogenic *Escherichia coli*
Cm: chloramphenicol
FBA: flux balance analysis
GEM: genome-scale metabolic reconstruction
3-HPAA: 3-hydroxyphenylacetate
LB: Luria–Bertani
lb: lower bound
MLST: multilocus sequence typing
mmol gDW^−1^ h^−1^: millimole per gram dry weight per hour
ST: sequence type
