# Supplementary material for: Use of genome-scale metabolic reconstructions of avian pathogenic Escherichia coli (APEC) phylogroups for the identification of lineage-specific metabolic pathways
Source: Microb Genom. 2025 Sep 17;11(9):001490. doi: 10.1099/mgen.0.001490 (PMC12444784; doi:10.1099/mgen.0.001490)
Supplement: Uncited Supplementary Material 1. [file mgen-11-01490-s001.pdf]

Supplementary Figures 1-5 for:

Use of Genome Scale Metabolic Reconstructions of Avian Pathogenic *Escherichia coli* (APEC) phylogroups for the identification of lineage-specific metabolic pathways

Huijun Long, Jai W. Mehat, HuiHai Wu, Arnoud H. M. van Vliet, Roberto M. La Ragione

Figure S1. Summary of the deleted reactions for construction of phylogenetic group specific sub-models based on APEC model.

Figure S2. The distribution of essential genes predicted by *in silico* analysis against glucose and glycerol as sole carbon sources, using the APEC model in different functional categories.

Figure S3. Scatter plots comparing computational predictions from the *E. coli* K-12 *i*JO1366 model and the APEC model for three APEC isolates representing phylogroups B2, C and G, with Biolog phenotypic observations with single nutrient source utilisation.

Figure S4. Growth of five representative Phylogroup C APEC isolates in M9 minimal media supplemented with 3-hydroxyphenylacetic acid (panels A-E) and in LB broth (panel F), respectively.

Figure S5. The growth curve of phylogroup B2 and G APEC isolates in M9 minimal media supplemented with 3-hydroxyphenylacetic acid (1 mM, 0.75 mM and 0.5 mM) and in LB broth, respectively.

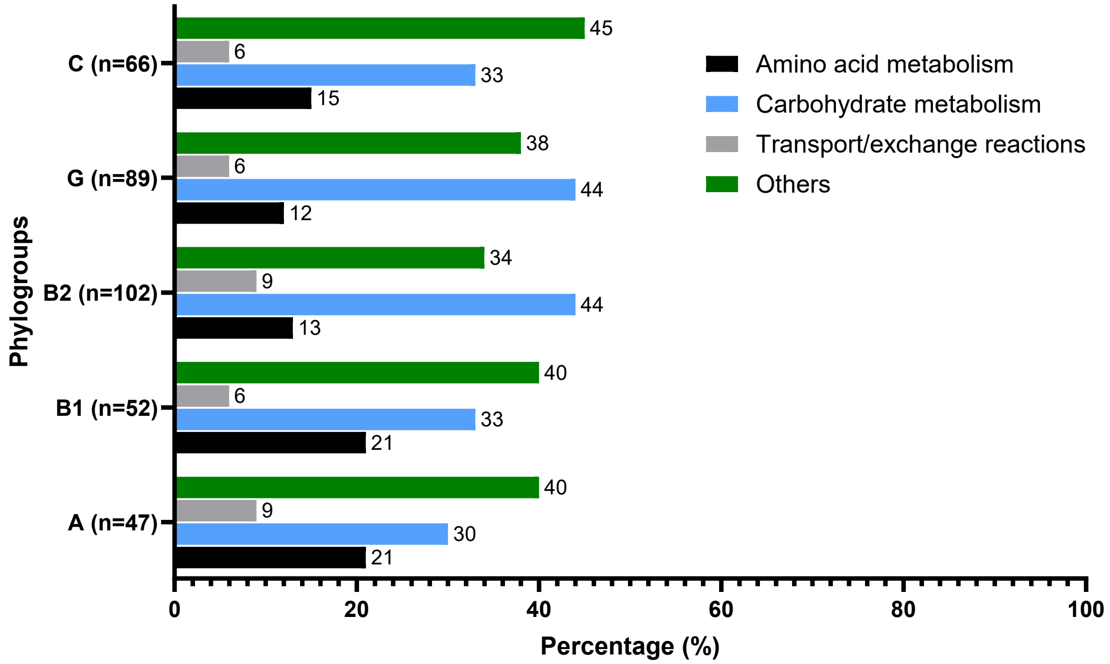

Figure S1. Summary of the deleted reactions for construction of phylogenetic group specific sub-models based on APEC model. A total of 133 reactions were removed in construction of phylogroups-specific sub-models for 114 APEC isolates in this study. Colour code of functional categories of reactions: Amino acid metabolism = Black; Carbohydrate metabolism = Blue; Transport/exchange reactions = Grey; Others* = Green. *Other reactions including Cell wall/ membrane/ envelope metabolism, Cofactor and prosthetic group metabolism, Energy production and conversion, Inorganic ion transport and metabolism, Lipid metabolism, Nucleotide metabolism and other described in Figure 2.

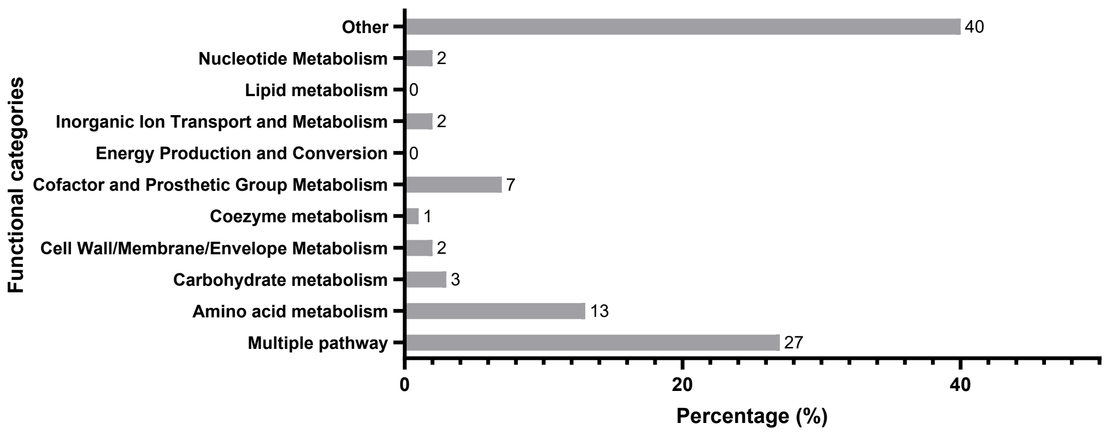

Figure S2. The distribution of essential genes predicted by *in silico* analysis against glucose and glycerol as sole carbon sources, using the APEC model in different functional categories.

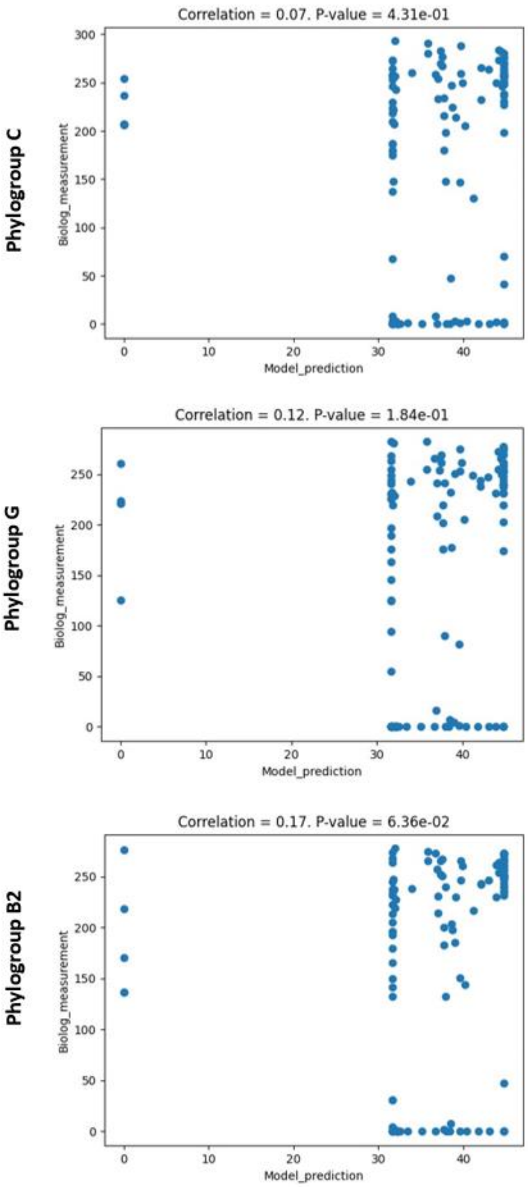

Figure S3. Scatter plots comparing computational predictions from the *E. coli* K-12 *i*JO1366 model and the APEC model for three APEC isolates representing phylogroups B2, C and G, with Biolog phenotypic observations with single nutrient source utilisation. The APEC isolates used are from phylogroup C (SAP0009), G (SAP0545) and B2 (SAP0474). The scatter plots show the model prediction (X-axis) using *i*JO1366a (Left) and APEC model (Right) and phenotypic observation using Biolog measurement (Y-axis). The correlation value shown above graphs is the r^2 value.

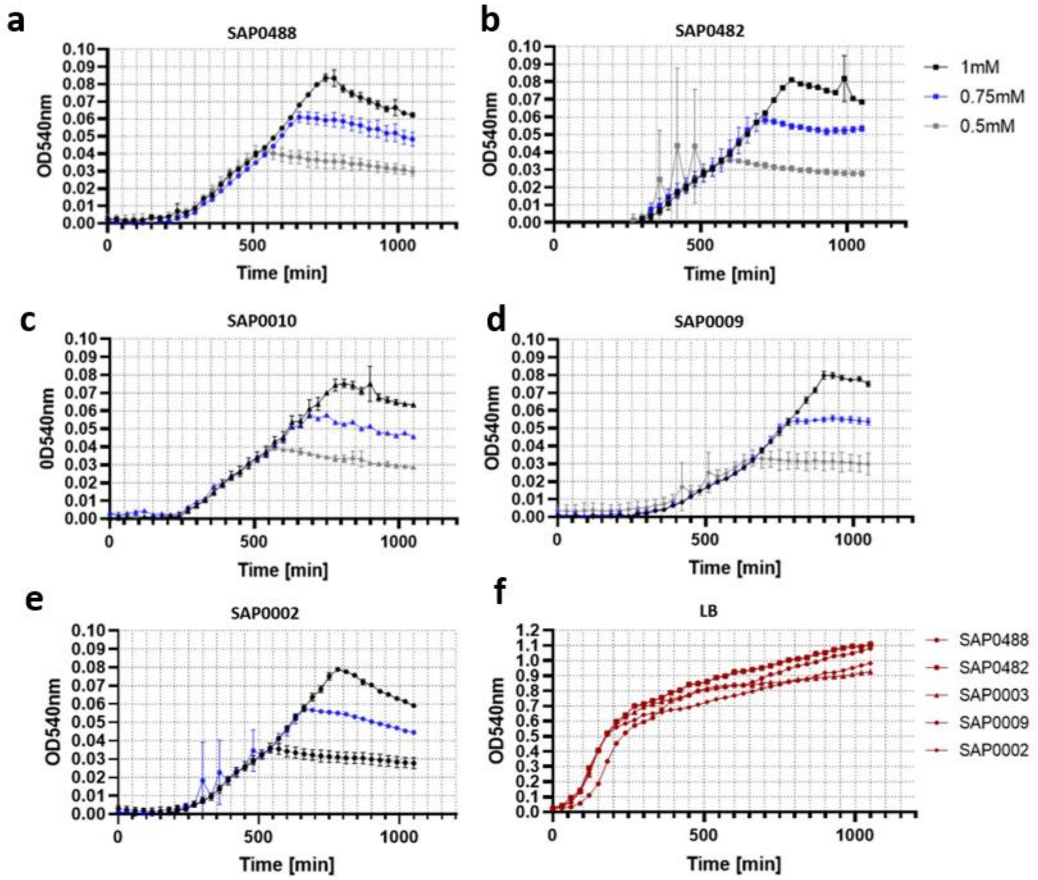

Figure S4. Growth of five representative Phylogroup C APEC isolates in M9 minimal media supplemented with 3-hydroxyphenylacetic acid (panels A-E) and in LB broth (panel F), respectively. The growth curves show the mean of 3 biological replicates, using optical density measurements at 600 nm wavelength for every 30 min in 18h, with error bars indicating the standard error of the mean (SEM). Panels A-E illustrates the growth curves of phylogroup C APEC isolates in M9 minimal media supplemented with three concentrations of 3-hydroxyphenylacetic acid (1 mM, 0.75 mM and 0.5 mM). Panel F shows growth curves of all group C isolates tested in this study in LB broth.

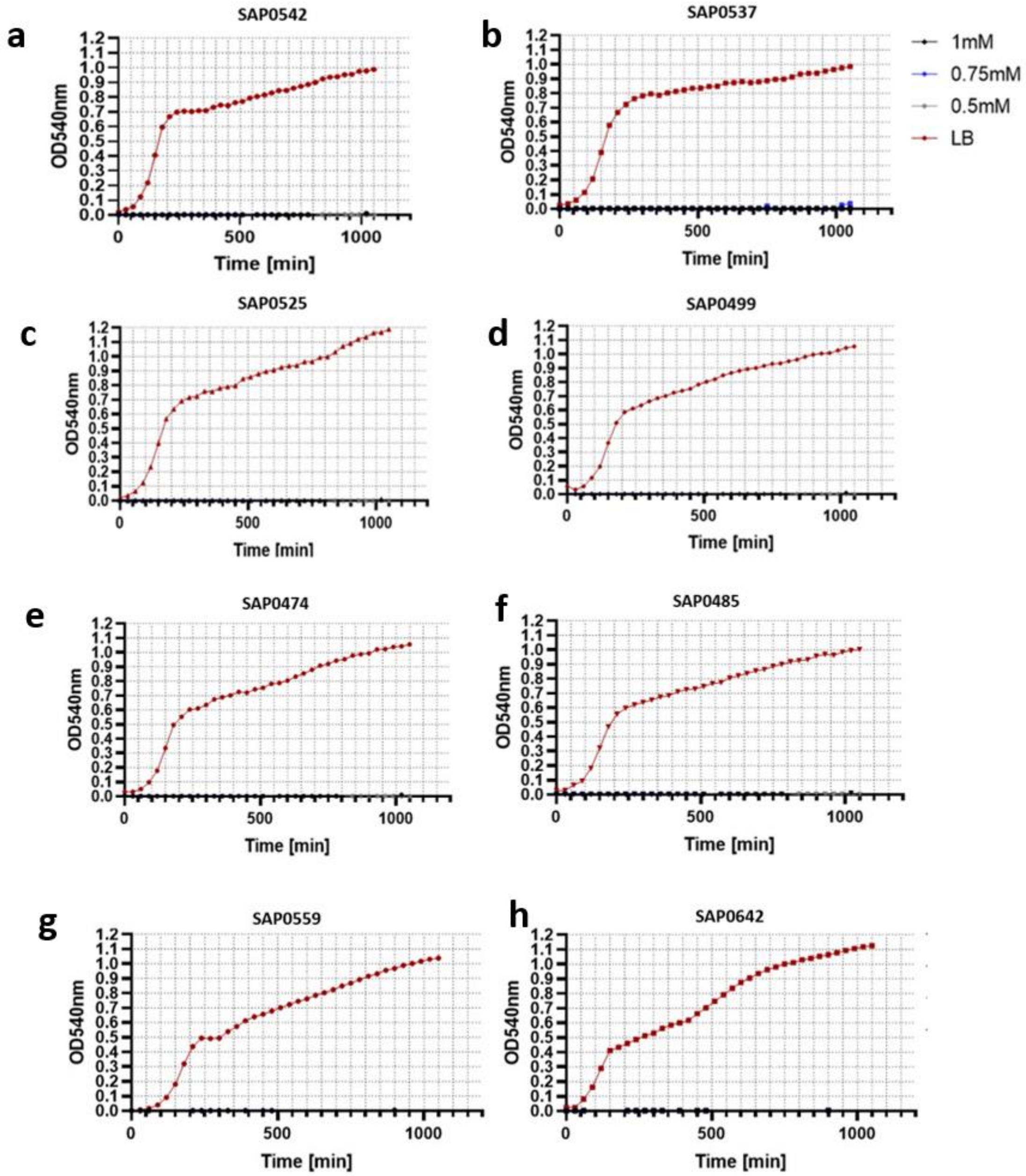

Figure S5. The growth curve of phylogroup B2 and G APEC isolates in M9 minimal media supplemented with 3-hydroxyphenylacetic acid (1 mM, 0.75 mM and 0.5 mM) and in LB broth, respectively. The growth curves show the mean of 3 biological replicates, using optical density measurements at 600 nm wavelength for every 30 min in 18h, with error bars indicating the standard error of the mean (SEM). Panels A-F shows the phylogroup B2 APEC isolates used in this study whilst panels G-H shows the phylogroup G APEC isolates used in this study. There was no growth observed with any of the phylogroup B2 and G isolates with any of the concentrations of 3-HPAA added.
